# The co-culture of ASCs and EPCs promotes vascularized bone regeneration in critical-sized bone defects of cranial bone in rats

**DOI:** 10.1186/s13287-020-01858-6

**Published:** 2020-08-03

**Authors:** Yuanjia He, Shuang Lin, Qiang Ao, Xiaoning He

**Affiliations:** 1 Department of Stomatology, The Fourth Affiliated Hospital of China Medical University, Shenyang, Liaoning China; 2 Department of Plastic Surgery, Shengjing Hospital affiliated to China Medical University, Shenyang, Liaoning China; 3 Department of Tissue Engineering, School of Fundamental Science, China Medical University, Shenyang, Liaoning China

**Keywords:** ASCs, EPCs, Osteogenesis, Vascularized bone regeneration

## Abstract

**Background:**

The repair of critical-sized bone defect represents a challenging problem in bone tissue engineering. To address the most important problem in bone defect repair, namely insufficient blood supply, this study aimed to find a method that can promote the formation of vascularized bone tissue.

**Method:**

The phenotypes of ASCs and EPCs were identified respectively, and ASCs/EPCs were co-cultured in vitro to detect the expression of osteogenic and angiogenic genes. Furthermore, the co-culture system combined with scaffold material was used to repair the critical-sized bone defects of the cranial bone in rats.

**Results:**

The co-culture of ASCs/EPCs could increase osteogenesis and angiogenesis-related gene expression in vitro. The results of in vivo animal experiments demonstrated that the ASC/EPC group could promote bone regeneration and vascularization in the meantime and then significantly accelerate the repair of critical-sized bone defects.

**Conclusion:**

It is feasible to replace traditional single seed cells with ASC/EPC co-culture system for vascularized bone regeneration. This system could ultimately enable clinicians to better repair the defect of craniofacial bone and avoid donor site morbidity.

## Background

The repair of critical bone defects represents a challenging problem in bone tissue engineering [1]. At present, bone tissue engineering technology has made great progress, but there are still some obstacles, such as the inability of osteoblasts to penetrate into the center, the limited amount of bone formation, the difficulty to construct large-scale bone tissue, and slow speed of ossification [2–5]. It is mainly due to the lack of angiogenesis in the implanted tissue-engineered bone, inability to obtain sufficient nutrition, reducing the amount of regenerated bone formation, and limiting the repair of critical bone defects [6]. It was confirmed in the previous studies that angiogenesis is an important factor that affects the osteogenesis process in vivo and capillary vessels are involved in the process of intra-membrane osteogenesis and endochondral ossification [7, 8]. At the site of new bone formation, osteoblasts and bone progenitor cells are found near vascular endothelial cells, suggesting that angiogenesis and osteogenesis are interdependent [9]. Meanwhile, in the process of osteogenesis, restricted angiogenesis will lead to poor bone integration and reduced mechanical strength [10]. In the repair of bone defect below the critical-sized defect, bone tissue has very strong regeneration ability and does not form fiber healing. This regenerative ability is achieved by relying on the vascular system around the defect area, which can continuously recruit nearby bone progenitor cells for osteogenic repair and provide nutritional support for the repair process. However, in the case of critical-sized defect, the obvious damage to the vascular system around the defect affects the recruitment of nearby bone progenitor cells and the transportation of nutrients, resulting in the limitation of the regeneration process of the bone tissue and the failure to complete the repair of the critical bone defect [11]. Therefore, in the process of repairing critical bone defects, it is very necessary to achieve vascularized bone regeneration [12]**.**

The application of endothelial progenitor cells (EPCs) is a potential method to achieve vascularization [13]. Endothelial progenitor cells (EPCs) are precursor cells of vascular endothelial cells, which have the ability to proliferate, migrate, and differentiate into cells arranged along the lumen of blood vessels [14]. It has been found in previous studies that EPCs can enhance the osteogenic activity of pre-osteoblasts [15] and play an important role in bone formation and repair [16, 17]. Adipose-derived mesenchymal stem cells (ASCs) are extracted from adipose tissue with high proliferative capability and multi-differential potential [18, 19]. Compared with stem cells derived from other tissues, ADSCs have the unique advantages of a wide range of tissue sources, easy availability, and small damage to donor site [20]. Some studies have demonstrated that ADSCs can secrete vascular endothelial growth factor (VEGF) through the paracrine pathway and promote the formation of new blood vessels [18, 21]. Therefore, the co-culture of ADSCs and EPCs may promote the formation of new bone and blood vessels through cell-cell interactions.

To study the effect of ADSC and EPC co-culture on the construction of vascularized bone tissue, our team first identified the phenotypes of ASCs and EPCs, and then determined whether co-culture of ASCs and EPCs could increase the differentiation of osteoblasts and vascular endothelial cells in vitro. We further used the ASC/EPC co-culture cell system combined with scaffold materials to repair severe bone defects in the cranial bone of rats and analyzed the results. The above results showed that the co-culture of ASCs and EPCs facilitate bone regeneration and angiogenesis and significantly promote the repair of critical-sized bone defects. It is feasible to use ASC/EPC co-culture cell system instead of traditional single seed cells for bone tissue engineering.

## Materials and methods

### Preparation and culture of ASCs and EPCs

Animal procedures were conducted in accordance with the protocol approved by IACUC of China Medical University. Sprague-Dawley (SD) rats, 4-week-old, were euthanized by CO_2_. The subcutaneous white adipose tissue of the inguinal region was cut off, while the blood vessels and other tissues were removed. The fresh tissue was washed three times with PBS containing 1% penicillin/streptomycin and minced by sterile surgical scissors. The minced tissue was digested with 0.1% collagenase type I (Gibco) at 37 °C for 1 h and then centrifuged at 1000 rpm for 5 min. The upper layer of fat was removed; the remaining cell pellet was resuspended and filtered through a 70-μm filter. After another centrifugation for 5 min, the cell pellet was resuspended in the culture medium consisting of low-glucose Dulbecco’s Modified Eagle Medium (DMEM) (Hyclone), 10% fetal bovine serum (FBS) (Gibco), and 1% penicillin/streptomycin. The suspension was transferred to a flask and cultured at 37 °C with 5% CO_2_ in a humidity atmosphere [22]. The culture medium was replaced 24 h after transferring into flasks [23]. The medium was replenished every 2–3 days, and the cells were passaged after 80% confluence. The ASCs at passage 3 were used for the following experiment.

After 4-week-old SD rats were sacrificed with CO_2_, their femurs were taken, and the bone marrow was evenly beaten. Single-cell suspension was obtained by centrifugation with Histopaque-1083 (Sigma), and mononuclear cell layer was separated by mononuclear cell separation solution. CD34+ cells were separated by immunomagnetic bead method. Cells were resuspended in EGM medium (Lonza, Cologne, Germany) and seeded into culture flasks and cultured at 37 °C with 5% CO_2_ in a humidity atmosphere. The culture medium was replaced 24 h to remove nonadherent cells.

The medium was replenished every 2–3 days, and the cells were passaged after 80% confluence. The EPCs at passage 3 were used for the following experiment.

### Flow cytometry analysis and immunofluorescence staining

1 × 10^6^ ASCs and EPCs at passage 3 were harvested, washed with 10% FBS/PBS, and centrifuged at 1000 rpm, 5 min to gather cell pellets. For flow cytometry analysis, ASCs were stained with FITC-conjugated rat anti-CD70, Cy5.5-conjugated rat anti-CD90, PE-conjugated rat anti-CD45, and Alexa Fluor 647-conjugated rat anti-CD34 antibodies at a concentration of 2 mg/ml at 4 °C [24]. EPCs were stained with PE-conjugated rat anti-CD31, Alexa Fluor 647-conjugated rat anti-CD34, FITC-conjugated rat anti-CD45, and FITC-conjugated rat anti-CD133, at a concentration of 2 mg/ml at 4 °C. Mouse IgG was served as negative controls. Processed specimens were washed with 2 ml of 10% FBS/PBS for 30 min. After resuspension in 500 μl PBS, the cell pellets were tested by flow cytometry with 10,000 events recorded for each condition. The results were analyzed by FACS Express software. For immunofluorescence staining, EPCs at passage 3 were co-stained with DPBS-E containing 10 mg/ ml DiI-labeled acLDL (Biomedical Technologies) for 60 min at 37 °C, and then observed under fluorescence microscopy.

### Co-culture of ASCs and EPCs in vitro

To determine the optimal ratio of EPCs and ASCs in bone regeneration, six groups were divided for experimental observation, namely ASCs alone, EPCs alone, and EPCs to ASCs at ratios of 1: 1, 1: 2, 1: 5, and 1:10. Cells were seeded in 12-well plates at the density of 1 × 10^5^ cells per well and induced with EGM/CM media (EGM media to complete media ratio of 1:1) or EGM/OS media (EGM media to OS media ratio of 1:1) for 7 days, which was prepared for ALP activity assay as previously reported.

### Alkaline phosphatase activity assay

ALP activity was detected by using an ALP assay kit (Sigma) following the manufacturer’s instructions. In brief, cells were mixed with an alkaline buffer solution (1.5 M, pH 10.3) containing 10 mM p-nitrophenyl phosphate as a substrate and NaOH solution (3 M) was used as stop solution. The optical density was measured at 405 nm with a microplate reader. ALP activity was normalized by the DNA content and expressed as nmol of p-nitrophenol produced per minute per mg of total DNA. The implanted samples were smashed in liquid nitrogen and lysed in 1 ml harvest buffer for 1 h, and then homogenized carefully to further lyse cells. After a centrifugation at 2000 rpm for 10 min, 10 ml supernatant were harvested for ALP activity assay.

### Quantitative reverse transcription-polymerase chain reaction (qRT-PCR)

Total RNA was isolated using Trizol reagent (Invitrogen) according to manufacturer’s instructions. Reverse transcription of total RNA was performed by RT-PCR (Invitrogen) using the reverse transcription first chain synthesis system. Real-time PCR reaction was performed with synthetic cDNA. Specific primers were used for PCR amplification to analyze the expression of osteoblastic marker genes including OCN, Col1a1, BMP2, vascular endothelial growth factor (VEGF), Cadherin5 (cdh5), and von Willebrand factor (vWF). According to the manufacturer’s instructions, real-time PCR was performed using SYBR GREEN PCR Master Mix on ABI PRISM 7500 sequence detection system. PCR conditions were 94 °C 1 min, 95 °C 30 s, and 58 °C 40 s, with a total of 35 cycles. All reactions were repeated three times and normalized to GAPDH. Comparative ct was used to calculate the relative difference of PCR results (Table 1).

**Table 1:** The primer sequences used for qRT-PCR

	Forward sequence	Reverse sequence
OCN	5′-tctttctcctttgccggc-3’	5′-caccgtcctcaaattctccc-3’
Col1a1	5′-gcaacagtcgcttcacctaca-3’	5′-caatgtccaagggagccacat-3’
BMP2	5′-tccgctccacaaacgagaaa-3’	5′-aaaggcatgatagcccggag-3’
VEGF	5′-ccgaaaccatgaactttctgc-3’	5′-gacttctgctctccttctgtc-3’
cdh5	5′-ggcaatcaa ctgtgctctcc-3’	5′-cttcgtgga ggagctgatct-3’
vWF	5′-ccggaagcgaccctcaga-3’	5′-cgg tcaattttgccaaagatct-3’

### von Kossa staining

After ASCs/EPCs were induced with osteogenic medium for 3 weeks, the formation of mineralized nodules in vitro was detected by von Kossa staining. Briefly described below, 1% silver nitrate solution was added to the culture medium for 45 min under ultraviolet light, rinsed with distilled water, and treated with 3% sodium thiosulfate for 5 min. After another rinse, specimens were re-stained and washed with ethanol, and then we finally performed microscopic observation, image acquisition, and analysis.

### Matrigel tubule formation assay

Twenty-four hours before the experiment, the Matrigel (BD Corporation, USA) was moved from − 20 °C to 4 °C refrigerator to fully melt. Fifty microliters of Matrigel was added to each well in a 96-well plate and placed in a 37 °C, 5% CO_2_ incubator for 2 h to coagulate. Cells of different groups were seeded to the 96-well plate containing Matrigel (5 duplicate wells per group), placed in the incubator, and observed under a microscope every 3 h. When there was obvious blood vessel formation, photographs were taken immediately. Image-Pro Plus 6.0 software was used to analyze the number and relative length of each component tube.

### Fabrication of HA/Col scaffold

According to a previous method of thermally induced phase separation [25], the scaffold preparation protocol is briefly described as follows. One gram of hydroxyapatite (HA) (Sangon Biotech, Shanghai, China) powder was dissolved into 10 ml of suspension with deionized water. After stirring at room temperature (RT) for 5 h, the Ha powder was fully dispersed with ultrasonic. Then, 40 ml (5 mg/mL) collagen (Kele Biological Technology Co. Ltd., Chengdu, China) solution was mixed with the Ha solution and stirred at RT for 2 h to form a Ha-collagen mixture (HA to Col = 8:2). Subsequently, the mixture was transferred to a circular mold with a diameter of 8 mm and a thickness of 3 mm, pre-frozen at a temperature of − 40 °C for 24 h, and then freeze-dried at a temperature of − 55 °C using a constant temperature freeze dryer.

### Scanning electron microscope (SEM)

Field emission SEM S-4800 (Hitachi, Japan) was employed to observe the microstructure of the HA/Col scaffold, as well as the morphology and behavior of the cells grown in the scaffold. The co-cultured ASCs/EPCs were inoculated on HA/Col scaffolds. After 3 days of culture, the cells were fixed with 2.5% glutaraldehyde fixation, and the samples were dehydrated with ethanol in grades. After dehydration, gold spraying was performed for 15–20 s. All samples were analyzed at 1.0 kv.

### Rat critical-sized cranial bone defect model

The in vivo experimental protocol was approved by IACUC of China Medical University. Thirty-two male SD rats, 8 weeks old, underwent surgery under general anesthesia, induced by 5% isoflurane/O_2_ gas inspiration and maintained by 1–2% isoflurane/O_2_ by a facial mask. The scalps were shaved cleanly, disinfected with iodophors, and infiltrated with 0.1–0.5 ml of a local anesthetic agent of 2% lidocaine with epinephrine (0.01 mg/ml). The skin and periosteum were incised along the midline to expose the cranial bone surface [26]. A trephine bur with diameter of 8 mm was used to create a standardized, round, segmental defect around the sagittal suture, maintaining the underlying dura mater intact. A single implant of 1 × 10^6^ cells mixed with the scaffold was inserted into each defect. The periosteum and skin were sutured in layers with non-absorbable 4–0 prolene sutures. For 2 days after surgery, the rats were treated with carprofen for analgesia and penicillin for prevention of infection. Animals were divided into 4 groups randomly: group 1, blank group; group 2, hydroxyapatite/collagen scaffold only (HA/Col); group 3, HA/Col +ASCs; and group 4, HA/Col +ASCs+EPCs. At the end of the eighth week after surgery, animals were euthanized with CO_2_ and the repaired calvaria bones were harvested for the following analyses.

### Analysis of bone regeneration

Micro-CT (Latheta LCT200) was used to scan the harvested samples for 3D imaging analysis. Meanwhile, bone mineral density (BMD, g/cm^2^) was performed with LUNAR PIXImus bone densitometer and analyzed by LUNAR PIXImus software according to the manual book of the equipment. A total of 6 samples were analyzed in each group. On the computerized scan, 5 regions of interest (ROI) of each slide were selected to measure the BMD of the defect area, and the average of these values was taken as the final result.

For histological analysis, six samples per group were decalcified and cut into 5 mm sections, half of which were used for hematoxylin-eosin (H&E) staining and the other half for immunohistochemistry analysis. Digital images of each slide were acquired using a digital camera mounted to a microscope. Newly formed bone areas in the total defect area were calculated manually at × 10 magnification by using NIH ImageJ software.

### Analysis of blood vessel ingrowth

VEGF was detected by immunohistochemistry in paraffin embedded and decalcitonized bone sections. As previously mentioned, VEGF is largely present in the subendothelial matrix. Half of the samples in each group were analyzed independently. The primary antibody of goat VEGF (Santa Cruz Biotechnology, Santa Cruz, CA) was diluted at a ratio of 1:300, and the hrp-conjugated rabbit anti-goat antibody (Jackson ImmunoResearch Laboratories, Inc. West Grove, PA) was diluted at a ratio of 1:500 to 1% BSA. The peroxidase activity was observed with diaminobenzidine. Both the negative control group and the positive control group were included under the same conditions. Negative control staining was performed on the same bone plates without primary antibodies. The kidney sections as positive group were stained with the same primary and secondary antibodies. AxioImager software was used for image acquisition. Using the NIH Image J software, the number of blood vessels in the entire implant area of each sample displayed by VEGF staining was calculated manually in a × 10 magnification manner.

### Statistical analysis

The test results were expressed as mean ± standard deviation, and statistical analysis was performed by SPSS 22.0 software (IBM SPSS Statistics, IBM, Armonk, NY). *T* test was used for the comparison of the data of two groups and one-way ANOVA was for the data of multiple samples. *p* < 0.05 was considered as statistically significant.

## Results

### Characterization of ASCs and EPCs

**Fig. 1:**
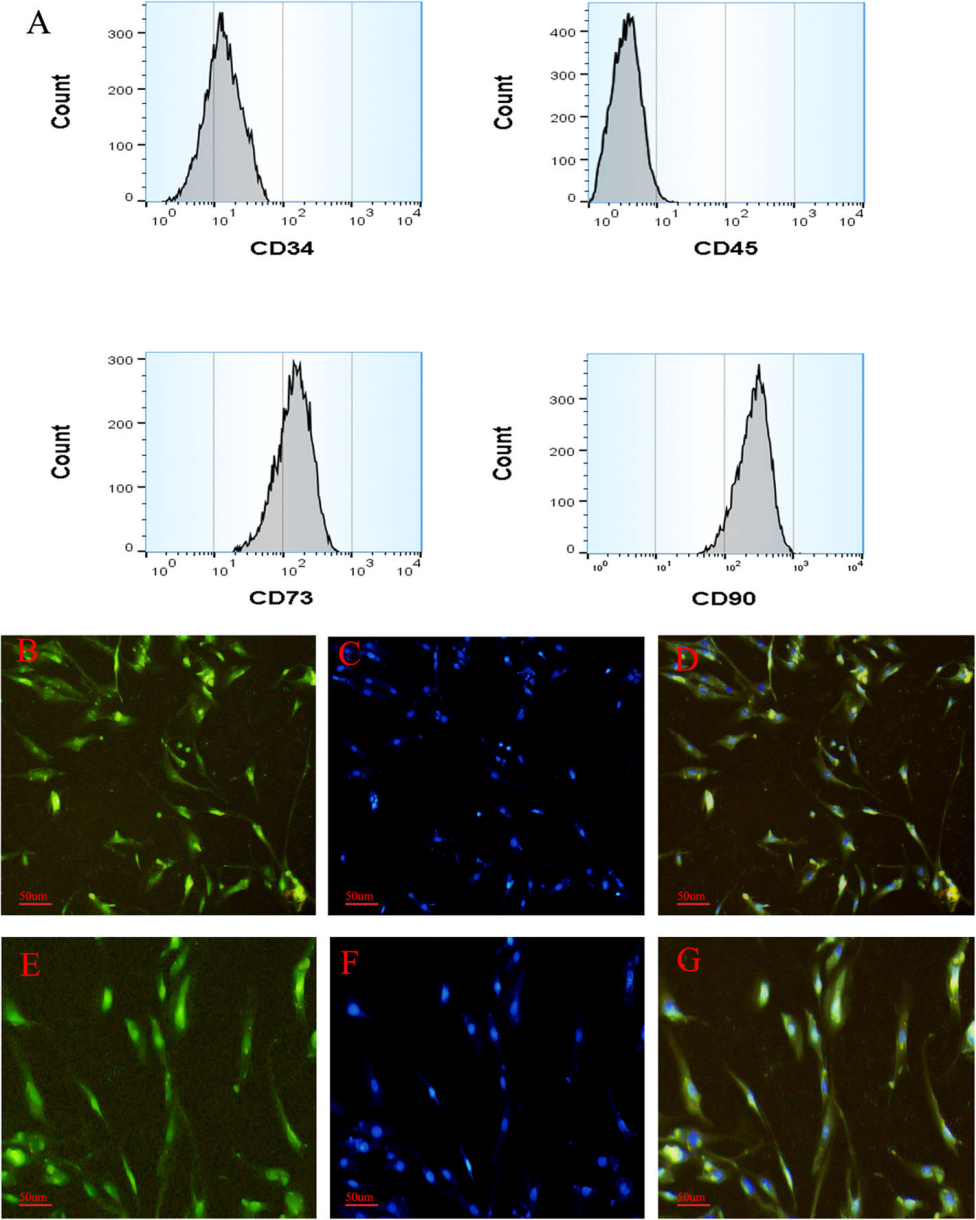
Surface markers of ASC flow cytometry (**a**). ASC immunofluorescence staining (**b–g**). **b** CD73. **c** DAPI. **d** CD73 + DAPI. **e** CD90. **f** DAPI. **g** CD90+DAPI. It was shown that the ASC markers CD73 and CD90 are positive

**Fig. 2:**
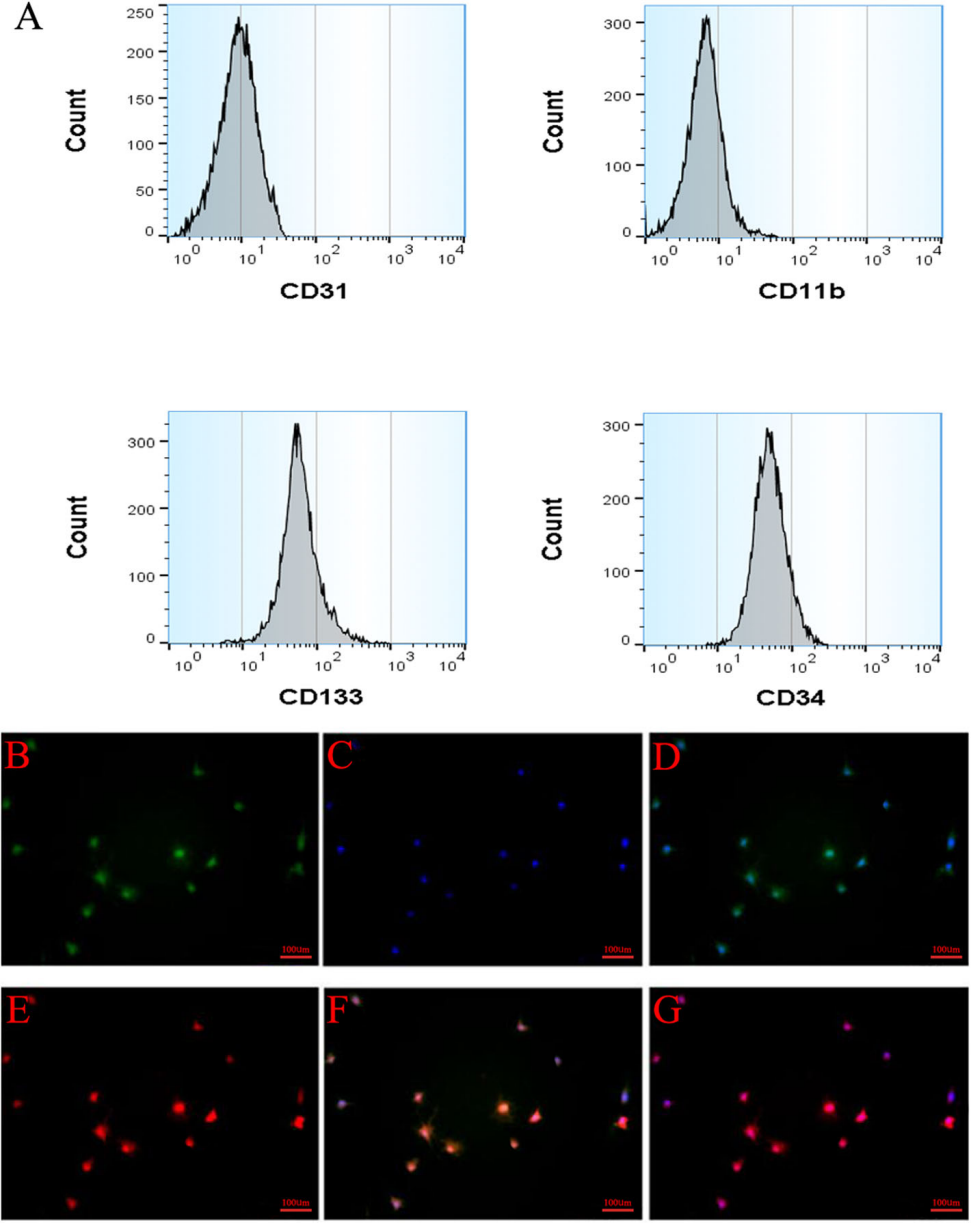
Surface markers of EPCs flow cytometry (**a**). EPC immunofluorescence staining (**b–g**). **b** VEGF. **c** DAPI. **d** VEGF+DAPI. **e** Dil-ac-LDL. **f** Lecin. **g** Merge of **e** and **f**

A number of surface proteins have been used to enrich rat ASCs and EPCs, including CD73, CD90, CD105, CD45, CD34, CD133, CD11b, and CD31. In our study, we used CD73, CD90, CD45, and CD34 as positive markers to enrich ASCs. The hematopoietic stem cells marker CD34, CD133, CD11b, and CD31 was used to identify EPCs. As shown in Fig. 1a, ASCs are positive for CD73 and CD90 and negative for CD45 and CD34. Meanwhile, the immunofluorescence staining results of ASC cell surface markers CD73 and CD90 (Fig. 1b–g) also suggested that CD73 and CD90 were positive. The flow cytometry results of EPCs are shown in Fig. 2a, indicating that EPCs were positive for CD133 and CD34 and negative for CD11b and CD31. To confirm the EPC phenotype, Dil-ac-LDL and lectin staining of EPCs were performed after the cells were cultured for 7 days. As shown in Fig. 2b–g, Dil-ac-LDL (red) and lectin (green) staining both were positive.

### Co-culture of ASCs and EPCs enhances osteoblast differentiation

**Fig. 3:**
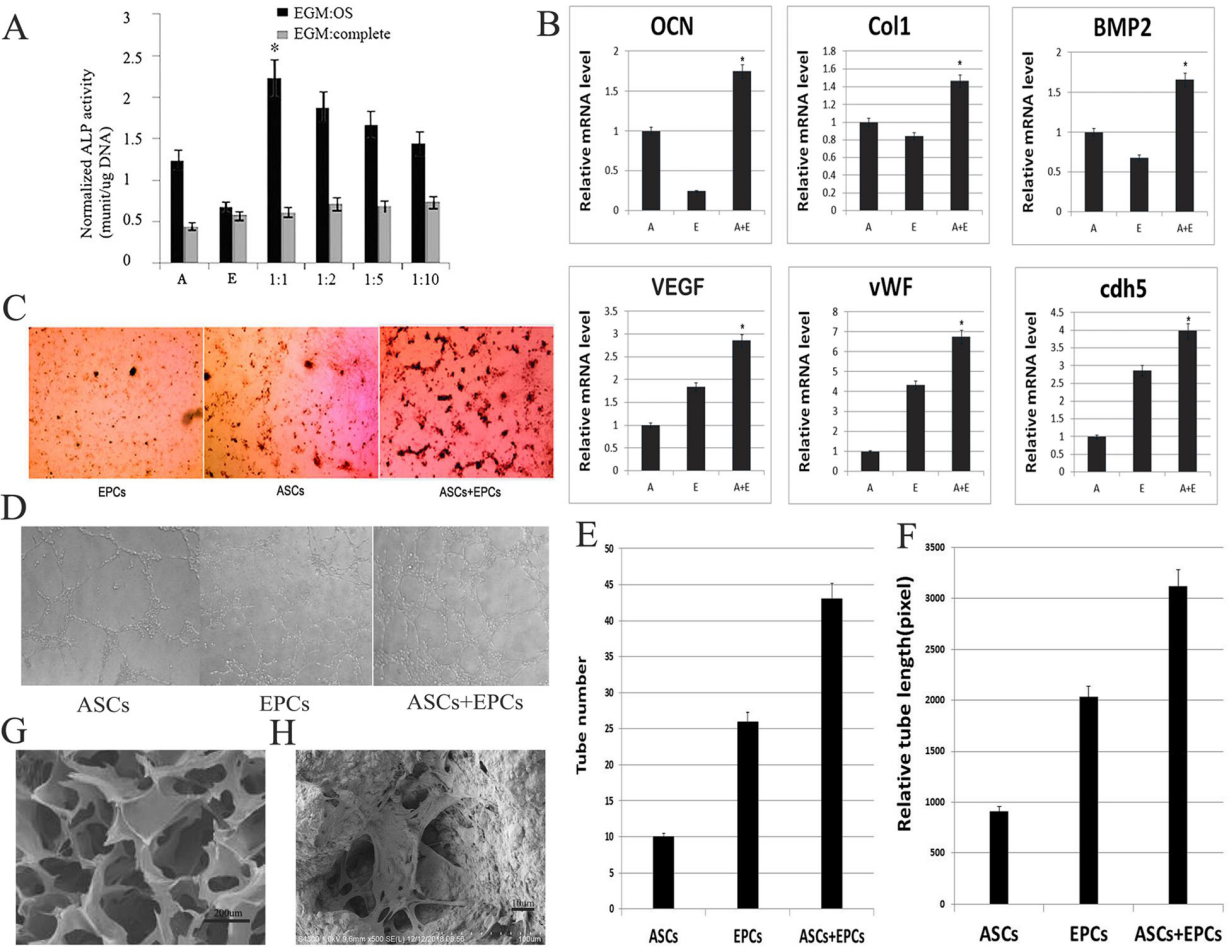
Analysis of results of co-culture of ASCs and EPCs in vitro. **a** Changes in ALP of ASCs and EPCs in different ratios (**a** ASCs, **e** EPCs). *p < 0.05. **b** RT-PCR detection of mRNA expression of osteogenic and angiogenic genes, osteogenic-related genes OCN, Col I, BMP2 and angiogenic-related genes VEGF, vWF, and cdh5 (**a** ASCs, **e** EPCs). *p < 0.05. **c** von Kossa staining. The number of calcified nodules in the ASC/EPC co-culture group was greater than that in the ASC and EPC groups. **d** The experimental results of Matrigel tubule formation assay under light microscope are presented. **e** Comparison of the number of tubular structures among the three groups, *p < 0.05. **f** Comparison of relative length of tubular structure among three groups, *p < 0.05. **g** The HA/Col void structure under scanning electron microscopy. **h** Cell growth morphology of ASCs/EPCs cocultured on HA/Col scaffolds in vitro

To identify the best ration for co-culture of ASCs and EPCs in osteoblast differentiation, ASCs and EPCs in different ratios were compared respectively for ALP activity after cultured with osteogenic induction medium and whole medium (Fig. 3a). The comparison results showed that the ALP activity of ASCs/EPCs at 1:1 ratio was significantly higher than that of other groups after induced by osteogenic induction culture. With the same co-culture ratio, the ALP activity of the osteogenic induction medium was higher than that of the whole medium group.

### Co-culture of ASCs and EPCs increases osteogenesis and angiogenesis-related gene expression

To further investigate whether the co-culture of ASCs and EPCs affected the expression of osteogenic and angiogenic genes, OCN, Col I, and BMP2 for osteogenesis and VEGF, cdh5, and vWF for angiogenesis were analyzed by using real-time PCR. The results showed that the expression levels of osteogenic genes OCN, Col I, and BMP2 in the ASC/EPC group were significantly higher than those in the ASC or EPC groups (Fig. 3b), suggesting that the co-culture of ASCs and EPCs can increase the expression of osteogenesis-related genes. The mRNA levels of angiogenic genes, including VEGF, cdh5, and vWF, were also dramatically higher in the ASC/EPC groups than in the single-cell groups (Fig. 3b), suggesting that the co-culture can also increase the expression of angiogenesis-related genes. It was indicated in these results that co-culture of ASCs and EPCs can enhance both of osteogenesis and angiogenesis-related gene expression. After the osteogenic medium induction was continued for 3 weeks, the formation of in vitro mineralized nodules was detected by von Kossa staining. The results showed that the number of in vitro mineralized nodules of ASCs/EPCs was significantly increased and the osteogenesis ability was significantly enhanced (Fig. 3c).

The results of Matrigel tubule formation assay showed that the density of tube structure in the ASC/EPC co-culture group was higher than that in the ASC/EPC group (Fig. 3d). The number of tubular structures increased significantly in the ASC/EPC co-culture group compared with the ASC or EPC group alone, and the difference was statistically significant at *p* < 0.05 (Fig. 3e). The length of the tubular structure formed in the co-culture group was also significantly longer than that of the other two groups, and the difference of *p* < 0.05 was statistically significant (Fig. 3f).

The SEM results of the scaffold showed that the cells of the ASC/EPC co-cultured cells had good morphology after being cultured on the HA/Col scaffold (Fig. 3g). The growth and proliferation of ASC/EPC co-cultured cells could be seen in the pores of the scaffold material (Fig. 3h), which showed that the HA/Col scaffold had no cytotoxicity and could be used as a cell scaffold material for further in vivo studies.

### Co-culture of ASCs and EPCs promotes vascularized bone regeneration in critical-sized bone defects of cranial bone in rats

**Fig. 4:**
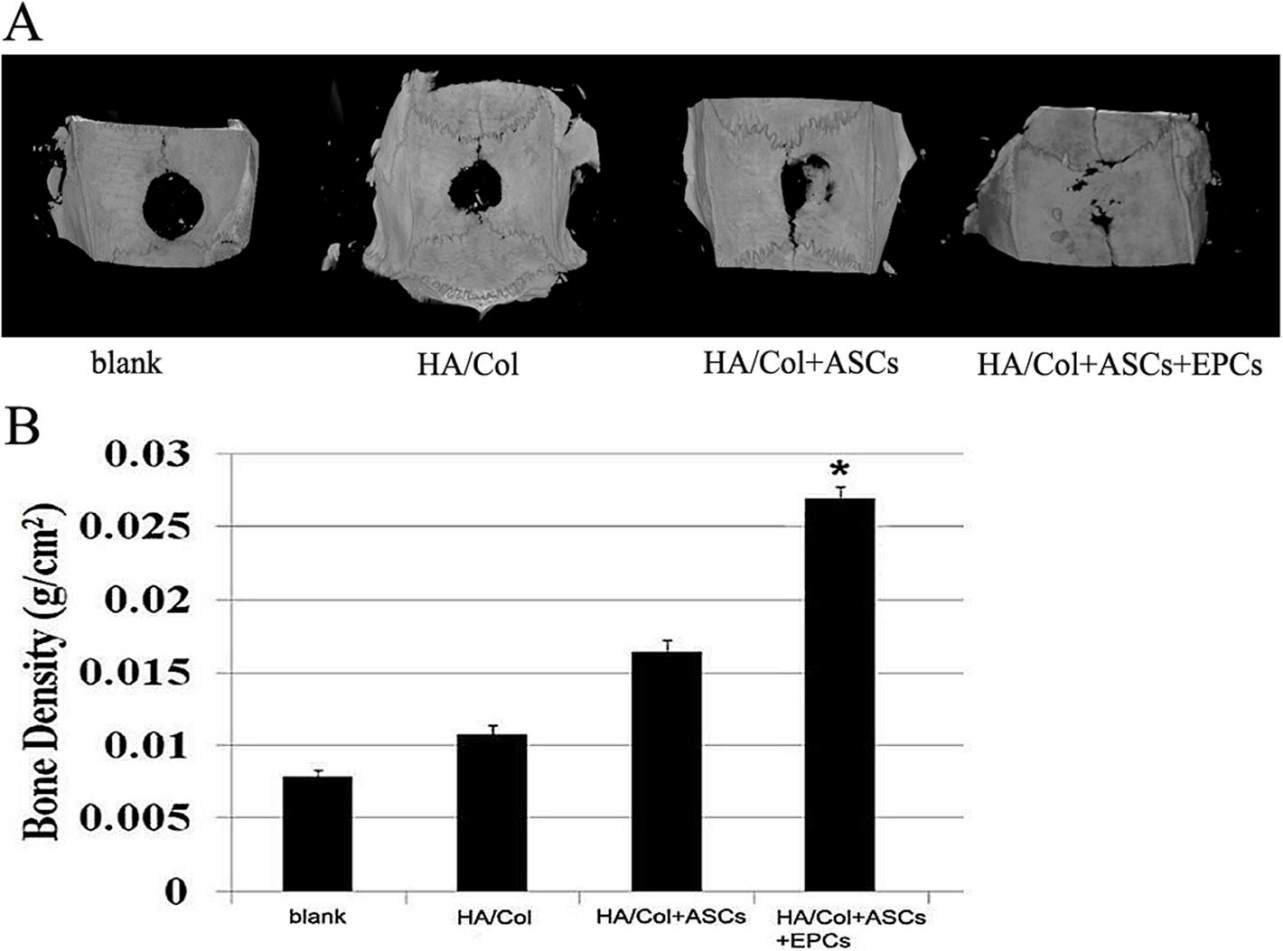
In vivo osteogenic effect analysis of skull defects in rats. **a** Three-dimensional Micro-CT imaging of the cranial bone at 8 weeks after surgery. **b** Quantitative analysis of BMD in the implantation area, *p < 0.05

To evaluate the potential of ASCs/EPCs for bone and vascular regeneration in vivo, 8-week-old SD rats with critical-sized bone defect (*d* = 8 mm) of the cranial bone were prepared as animal models. ASCs/EPCs were combined with hydroxyapatite/collagen (HA/Col) scaffolds to repair the critical-sized bone defects, and the repaired bones were harvested at the 8th week after surgery. As shown in the three-dimensional imaging by Micro-CT scan (Fig. 4a), group 4 (HA/Col+ASCs+EPCs) had remarkably stronger osteogenic activity. Compared with the other three groups, the bone defect area in group 4 was almost completely closed, which indicated that the co-culture of ASCs and EPCs enables the promotion of bone regeneration. Quantitative analysis of BMD showed that bone density in the implantation area of group 4 was dramatically higher than the other three groups (*p* < 0.05) (Fig. 4b).

**Fig. 5:**
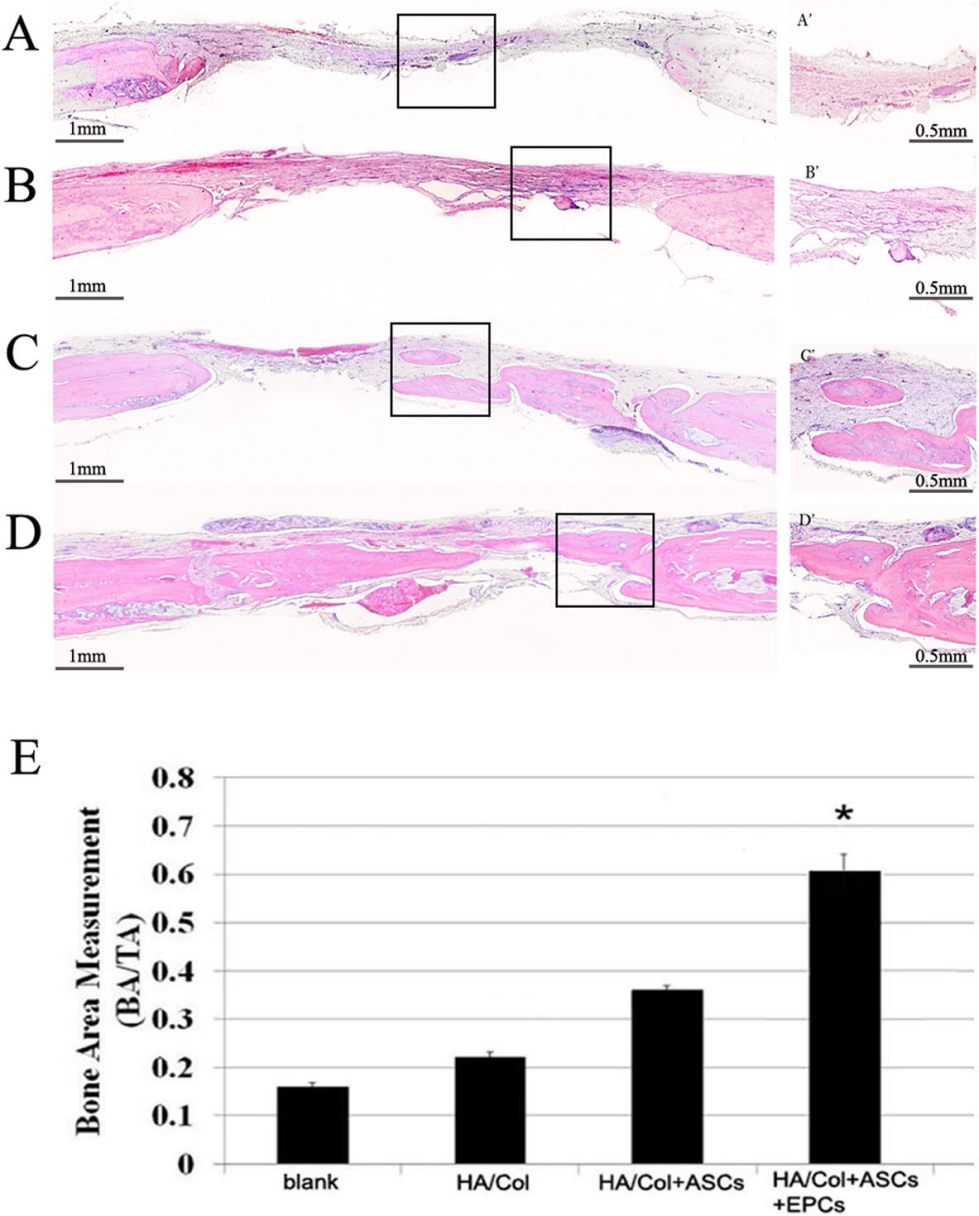
Hematoxylin and eosin staining analysis of new bone formation (**a**–**d** low magnification, bar = 1 mm; A’–D’ high magnification, bar = 0.5 mm. **a** A’ blank control group, **b** B’ HA/Col group, **c** C’ HA/Col+ASC group, **d** D’ HA/Col+ASC+EPC group). **e** Quantitative analysis of bone area in implanted region. BA, bone area in implant; TA, total implant area. *p < 0.05

HE staining sections showed no residual material or inflammatory infiltrating cells in the defect area after 8 weeks of implantation. In all samples, the amount of new bones in the blank and HA/Col groups was significantly smaller when compared with other groups, and most of the defect area was covered with fibrous tissue (Fig. 5a, b). In group 2 (HA/Col+ASCs), new bone was formed, partially covering the defect area, and still part of the defect area was covered by fibrous tissue (Fig. 5c), while group 4 showed robust osteogenic activity, and regenerated bone continuously covered almost all defect areas (Fig. 5d). Histomorphometric analysis showed that the amount of newly formed bone (BA) to the total implant area (TA) in group 4 was significantly greater than that in the other four groups (*p* < 0.05) (Fig. 5e), indicating that EPCs could promote bone regeneration.

### The ingrowth of blood vessels in the regenerated bone

**Fig. 6:**
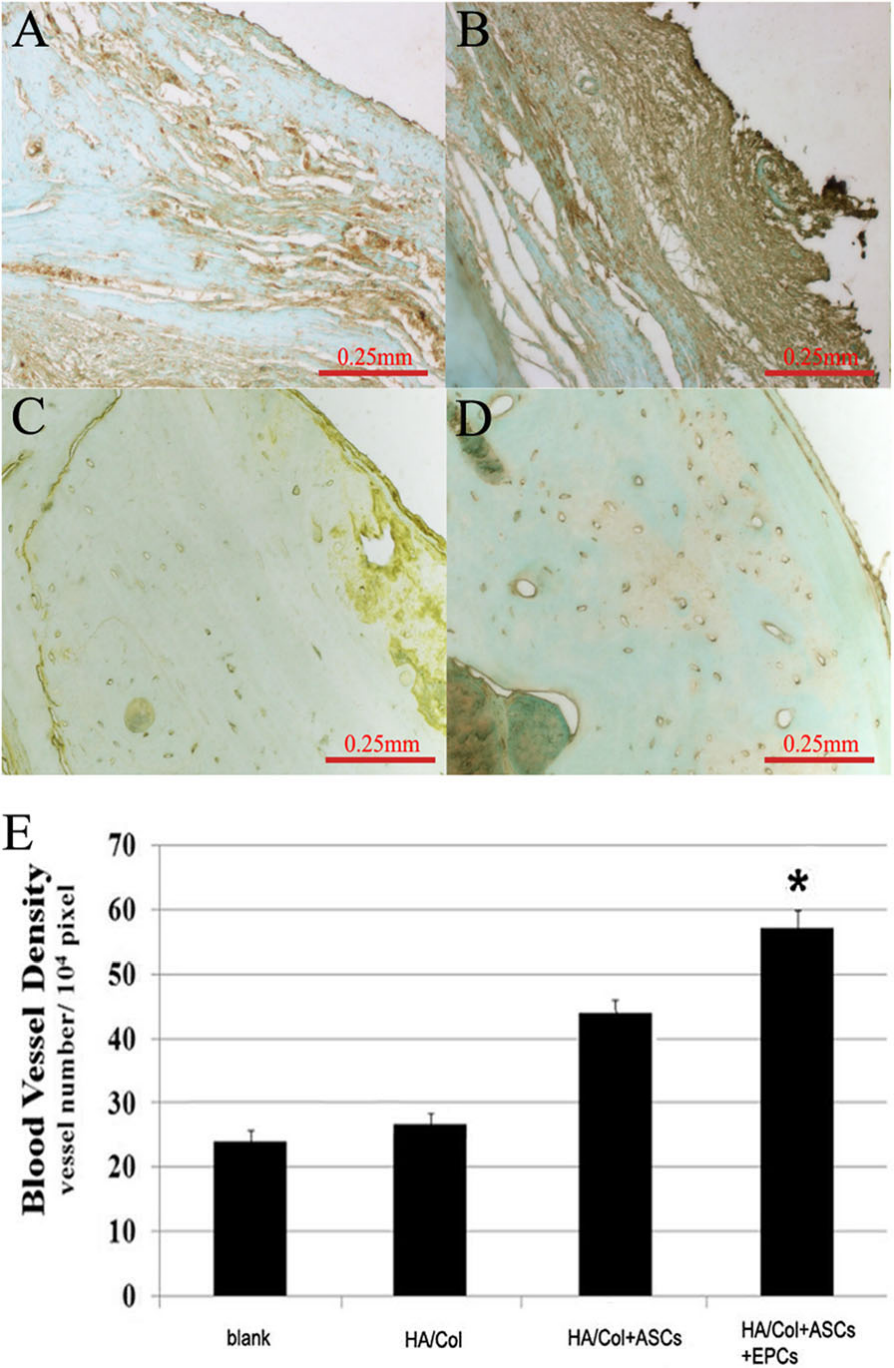
Immunostaining of VEGF in the newly formed bone (**a–d**) (**a** blank control group, **b** HA/Col group, **c** HA/Col+ASC group, **d** HA/Col+ASC+EPC group). **e** Quantitative analysis of blood vessel density. *p < 0.05

To confirm the formation of blood vessels within the implants, immunostaining with VEGF antibodies was performed, which could specifically stain and identify vascular endothelial cells. As shown in Fig. 6a–d, much more blood vessels were found in the HA/Col+ASC+EPC group compared to the other three groups. By quantifying the blood vessel density (BVD) of the entire implant area, it was confirmed that the BVD of the ASC/EPC group was significantly higher than that of the other groups (Fig. 6e). These results demonstrated that EPCs could promote vascular growth and that the co-culture of ASCs and EPCs dramatically enhances vascularized bone regeneration.

## Discussion

For the repair of critical-sized bone defect, the difficulties are mainly focused on the formation of vascularized bone, especially the center of the defect. To construct bone tissue accompanying vascular system, our team co-cultured ASCs and EPCs to establish a dual stem cell system. In this study, it was found that the ASC/EPC co-culture system can enhance the expression of osteogenic and angiogenic genes in vitro, and furthermore, by supporting vascularized bone regeneration, it can significantly accelerate bone healing of critical-sized bone defects in vivo.

EPCs are precursor cells of vascular endothelial cells, which have the ability to proliferate, migrate, and differentiate into cells arranged along the lumen of blood vessels and can be isolated from peripheral blood and spleen. ASCs also have high proliferative growth characteristics and multi-differential potential, can be extracted from autologous subcutaneous fat, and have a wide range of tissue sources. But to date, specific markers for each cell type are still lacking. A number of surface proteins have been used to identify rat adipose stem cells, including CD73, CD90, CD105, and CD44 [27–30]. Here, CD73 and CD90 are used as positive markers to identify ASCs. The results demonstrated that ASCs expressed a cell-surface protein profile positive for CD73 and CD90 and negative for CD45 and CD34. For EPCs, we detected the cell markers CD34 and CD133 [31], which are highly expressed in EPCs, but not expressed after the EPCs differentiated into mature vascular endothelial cells. Meanwhile, we also detected CD31, which is not expressed in EPCs, but highly expressed in mature endothelial cells, as well as CD11b, which is expressed in monocytes, but not in EPCs [32–35]. Our results showed that EPCs expressed a surface protein profile positive for CD133 and CD34 and negative for CD11b and CD31. These results confirmed the ASC and EPC phenotypes, suggesting that the above markers can be used for the identification of ASCs and EPCs.

In the design of the grouping of ASC/EPC co-culture ratios, we designed 6 groups of cell co-culture in vitro experiments with different ratios and grouped the cells in the co-culture with EPCs from small to large proportions. The purpose was to investigate the effect of different EPC ratios on the osteogenic effect, in order to determine the optimal co-culture ratio. It was found that when the ratio of ASCs/EPCs was 1:1, the ALP activity in the osteogenic inducing group is significantly higher than other groups, indicating that 1:1 ratio is more conducive to co-cultured cells for osteogenic differentiation. After determining the optimal ratio, real-time RT-PCR was performed to analyze the expression of OCN, Col I, and BMP2 of ASC osteoblast marker genes and VEGF, cdh5, and vWF of endothelial marker genes. The results showed that the ASC/EPC co-culture system could increase gene expression of both osteogenesis and angiogenesis. von Kossa staining was used to detect the formation of mineralized nodules in vitro, which also confirmed that ASC/EPC co-culture could improve the osteogenesis. In addition, it was indicated in the Matrigel tubule formation experiment that ASC/EPC co-culture could improve the angiogenic capacity of cell lines. In terms of scaffold materials, hydroxyapatite/collagen scaffolds were synthesized and observed using SEM. It was shown that ASC/EPC co-cultured cells could grow and proliferate on the scaffold materials with good morphology, proving that this scaffold does not affect cell activity and could be used as a bone regeneration scaffold material for the in vivo experiments.

Based on the in vitro results, we further performed an in vivo experiment on the repair of critical-sized bone defects in rats. According to previous researches, EPCs cannot be used alone to regenerate bone tissue, and excessive EPC transplantation even inhibits bone formation [36, 37]. The main purpose of this study is to observe the difference in vascularized bone formation between the ASC/EPC co-culture group and the ASC alone group. Therefore, four groups were designed in animal experiments: blank control group, scaffold group, scaffold+ASCs, and scaffold+ASCs/EPCs. The Micro-CT scans of the rat’s cranial bones were performed at 8 weeks after surgery. The results clearly showed that the amount of new bone in the defect area was much greater in the HA/Col+ASC+EPC group than in the other groups, including the HA/Col+ASC group. In addition, BMD also showed that there was more bone tissue formation in the HA/Col+ASC+EPC group. Moreover, as confirmed by immunohistochemical analysis, the blood vessel density in the defect area was higher in the HA/Col+ASC+EPC group than in the other three groups. The possible reason for this phenomenon is that the lack of EPCs in the ASC group resulted in a relative decrease in vascularization and bone formation, which relied solely on the inward growth of the host blood vessels. However, the distance between the blood vessels of the host tissue and the center of the bone defect is far from sufficient to achieve bone regeneration, especially for critical-sized defect. As a result, nutrients, metabolites, and other molecules cannot be delivered to the center area of defect, which severely impedes bone regeneration [38]. In the ASC/EPC group, EPCs directly increased the invasion of blood vessels and promoted the differentiation of ASCs to osteogenesis and, meanwhile, pre-osteogenic ASCs could promote the recruitment of EPCs and enhance the ability of EPCs to form blood vessels. The interaction accelerates vascularization and bone formation in the meantime, making nutrients, cytokines, and other molecular factors involved in the bone healing process more accessible.

The results also showed that the implanted EPC/ASC co-culture system had a synergistic response compared with the ASCs alone. In the co-culture group, bone tissue formation was significantly promoted, which may be the result of increased vascularization, leading to better recruitment of bone progenitor cells and other cells and molecular factors involved in bone healing. It has been widely accepted the close relationship between blood vessels and osteoblasts [39]. Vascularization is considered a necessary condition for bone formation, and insufficient vascularization can result in damaged bone formation or delayed bone healing. Studies have indicated that soluble cytokines produced by the co-culture of bone progenitor cells and endothelial cells could promote endothelial cell migration through autocrine or paracrine effects, leading to cell rearrangement and tubular network formation [40]. Vascular endothelial growth factor (VEGF) is considered to be the major promoter of endothelial cell migration and tubular network formation. Activation of VEGF induces phosphorylation, leads to transduction of different signals, promotes endothelial cell migration, and enhances angiogenesis [41]. Recent studies have shown that the combination of angiogenesis (VEGF) and osteogenic factor (BMP-2) promotes bone healing and regeneration, and endothelial cell transplantation improves bone marrow stromal cell-mediated bone regeneration in skull defect models [42, 43]. Regulation of cadherin signaling pathways may be involved in cell migration events in this co-culture system and combines with the release of soluble chemical attractants [44]. In this experiment, VEGF and its receptor were upregulated in the co-culture group, which might be due to the production of cytokines such as VEGF through autocrine and paracrine effects of ASCs, while VEGF could activate corresponding signaling pathways to promote vascular endothelial cell migration and formation of tubular structures, thereby increasing vascular formation. However, the molecular mechanism of this synergistic signaling pathway in the vascularized bone formation process of the EPC/ASC co-culture system is not yet clear, which is also the focus of the next research aim of this study team.

## Conclusion

In conclusion, this study provided evidence that the ASC/EPC co-culture cell system could synergistically promote vascularized bone formation, thereby achieving the repair of critical bone defects, which could not be achieved with other single seed cells. Moreover, the co-culture cell system, compared with stem cell angiogenesis gene transfection and other pro-vascularization methods, has the characteristics of a wide range of cell sources, easy availability, and low cost; therefore, it has good application prospects. On the basis of the data presented here, it was concluded that it is feasible to replace traditional single seed cells with ASC/EPC co-culture system as a potential way for vascularized bone regeneration.

## Data Availability

The datasets used and/or analyzed during the current study are available from the corresponding author on reasonable request.

## Abbreviations

EPCs: Endothelial progenitor cells
ASCs: Adipose-derived mesenchymal stem cells
BMP2: Bone morphogenic protein 2
VEGF: Vascular endothelial growth factor
Cdh5: Cadherin
vWF: Von Willebrand factor
HA/Col: Hydroxyapatite/collagen scaffold
BMD: Bone mineral density
BVD: Blood vessel density
HSCs: Hematopoietic stem cell
